# Protein aggregation containing beta-amyloid, alpha-synuclein and hyperphosphorylated tau in cultured cells of hippocampus, substantia nigra and locus coeruleus after rotenone exposure

**DOI:** 10.1186/1471-2202-11-144

**Published:** 2010-11-10

**Authors:** Rodrigo S Chaves, Thaiany Q Melo, Stephanie A Martins, Merari FR Ferrari

**Affiliations:** 1 Department of Neurology, School of Medicine, University of Sao Paulo, Sao Paulo, Brazil

## Abstract

**Background:**

Protein aggregates containing alpha-synuclein, beta-amyloid and hyperphosphorylated tau are commonly found during neurodegenerative processes which is often accompanied by the impairment of mitochondrial complex I respiratory chain and dysfunction of cellular systems of protein degradation. In view of this, we aimed to develop an *in vitro *model to study protein aggregation associated to neurodegenerative diseases using cultured cells from hippocampus, locus coeruleus and substantia nigra of newborn Lewis rats exposed to 0.5, 1, 10 and 25 nM of rotenone, which is an agricultural pesticide, for 48 hours.

**Results:**

We demonstrated that the proportion of cells in culture is approximately the same as found in the brain nuclei they were extracted from. Rotenone at 0.5 nM was able to induce alpha-synuclein and beta amyloid aggregation, as well as increased hyperphosphorylation of tau, although high concentrations of this pesticide (over 1 nM) lead cells to death before protein aggregation. We also demonstrated that the 14kDa isoform of alpha-synuclein is not present in newborn Lewis rats.

**Conclusion:**

Rotenone exposure may lead to constitutive protein aggregation *in vitro*, which may be of relevance to study the mechanisms involved in idiopathic neurodegeneration.

## Background

Intra and extracellular accumulation of protein aggregates distributed throughout the central nervous system are hallmarks of neurodegenerative diseases like Parkinson's (PD) and Alzheimer's (AD) [1] as well as are present in the senile brain [2].

Intracellular insoluble inclusions containing the alpha-synuclein protein, called Lewy bodies are commonly found in the brainstem, cerebellum, hypothalamus and autonomic nervous system of patients with PD, Lewy Body dementia, multiple system atrophy and other synucleinopathies [3-7].

Extracellular deposition of beta-amyloid peptide, derived from the abnormal cleavage of amyloid precursor protein, and intracellular neurofibrillary tangles of hyperphosphorylated tau protein are features of the hippocampus, cerebellum, locus coeruleus and cerebral cortex [8] of healthy elderly individuals [9] and patients with AD and other senile dementias [10].

Another common characteristic of neurodegenerative disorders is the impairment of mitochondrial complex I respiratory which may lead to *in vivo *protein aggregation [11].

In view of this, the present study aims to develop a method of *in vitro *aggregation of alpha-synuclein, hyperphosphorylated tau and beta-amyloid in cultured cells from the hippocampus, substantia nigra and locus coeruleus using treatment with rotenone, which is a natural pesticide and specific inhibitor of mitochondrial NADH dehydrogenase within complex I of the respiratory chain leading to increase in oxidative stress possibly mimicking what occurs during the ageing process [12,13]. The most characterized effects of rotenone is on mitochondrial complex I, however this compound is lipophilic being able to cross the cells membrane and to inhibit the proteasome [14], promote dysfunction in GAPDH [15] and interact also with glial cells [16].

## Methods

All procedures were performed in accordance to the institutional committee for animal care of the School of Medicine, University of Sao Paulo (#0659/08).

### Cell culture

Methodology employed for cell culture was a modification of the previously described protocol [17]. Briefly, neonatal Lewis rats had their brains dissected and the areas containing hippocampus, locus coeruleus and substantia nigra were excised. After dissection, blood and epithelial cells were removed in sterile cold solution consisting of NaCl 120 mM, KCl 5 mM, KH_2_PO_4 _1.2 mM, MgSO_4 _1.2 mM, NaHCO_3 _25 mM, glucose 13 mM, pH 7.22. Subsequently, the tissues were cut into small pieces using a scissors and incubated with 0.05% trypsin (Gibco) at 37°C for 40 minutes in a water bath kept under agitation. Then, trypsin inhibitor (0.006%, Gibco) was added and the cells were mechanically dissociated using a Pasteur pipette, after the total decoupling the cell solution was centrifuged at 300 g for 5 minutes. The supernatant was discarded and cells were resuspended in Neurobasal A medium (Gibco) supplemented with Glutamax (Gibco) 0.25 mM, B27 (Gibco) 2%, L-Glutamine (Sigma) 0.25 mM and Gentamicin (Gibco) 40 mg/l.

Cells were plated into either 8-well glass slides or 35 mm petri dishes (Nunc), at the concentration of 1800 cels/mm^2^. Plates were treated the day before with poly-_D_-lysine 10 μg/ml (Sigma), and with fetal bovine serum 10% (Gibco) for 2 hours before plating the cells to facilitate adhesion. Cultures were kept in a humidified incubator with 5% CO2 at 37°C for nine days. Culture medium was changed three hours after plating the cells and every three days of cultivation.

### Cell culture characterization

Cell cultures were washed in PBS, fixed in 50% methanol and 50% acetone for 10 minutes at -20°C, permeabilized with PBS containing 0.2% Triton for 30 minutes at room temperature. Unspecific binding sites were blocked with PBS containing 2% NGS (Vector Laboratories), 0.2% Triton and 4% bovine serum albumin (BSA, Sigma) for 30 minutes at room temperature.

Cells from substantia nigra and locus coeruleus were incubated with mouse polyclonal antibody against tyrosine hydroxylase (1/3000, Sigma) for 24 hours at 4°C, followed by incubation with anti-mouse immunoglobulin conjugated to FITC (Jackson, 1/120) for 45 minutes at room temperature protected from light. Hippocampal cultures were subjected to MAP2 immunolabeling (1/1000, Sigma) also at 4°C overnight followed by incubation with FITC-conjugated secondary antibody in order to identify the neurons present in cultures.

The slides were mounted with mounting medium containing DAPI (4',6-diamidino-2-phenylindole, Vector Laboratories) to visualize cell nuclei. Immunolabeled cells were analyzed using a fluorescence microscope (Zeiss) equipped with appropriated filters using a 40× lens. Quantification was done by comparing images taken of 16 fields of culture plate using filters to visualize the label generated by FITC and DAPI. Cell culture characterization was repeated twice.

### Exposure to Rotenone

Rotenone (Sigma, USA) was prepared with DMSO (Sigma, USA) (stock solution of 1 mM) and diluted in culture medium applied to cell cultures from hippocampus, locus coeruleus and substantia nigra in concentrations of 0.5, 1, 10 and 25 nM for 48 hours. Control groups were exposed to less than 0.01% DMSO diluted in culture medium. Cells were then subjected to trypan blue staining in order to identify cell death; to immunocytochemistry for identification of protein aggregates containing hyperphosphorylated tau, alpha-synuclein and beta-amyloid; and protein extraction for western blot experiments.

### Analysis of cell death

After exposure to rotenone, 10 μl of trypan blue stain solution (Gibco) which stains in blue the cytoplasm of cells with damaged plasma membrane, were added to the culture medium of cells. Immediately after the addition of trypan blue, the cells were examined under a microscope (Olympus) using an objective of 40× (400× magnification) and photographed to detect stained cells.

### Identification of protein aggregates through immunocytochemistry

Cell cultures were fixed as described above and incubated for 24 hours at 4°C with either a mouse polyclonal antiserum against alpha-synuclein (Abcam, 4D6, Ab1903), or rabbit polyclonal antiserum against hyperphosphorylated tau (Sigma, Ser 199/202, T6819) or beta amyloid peptide (Abcam, Ab14220), the three antibodies were diluted 1/1000 in PBS containing 0.3% Triton X-100 (Sigma) and 0.5% BSA (Sigma). Cells were washed in PBS and incubated with biotinylated goat anti-rabbit or anti-mouse immunoglobulin both diluted 1/200 (Vector, USA) for 2 hours at room temperature. Cells were washed in PBS and incubated with an avidin-biotin peroxidase complex (both diluted 1/120, Vectastain, Vector) for 2 hours. Immunoreactivity was visualized after 10 minutes of reaction with 3-3'-diaminobenzidine tetrahydrochloride (DAB, Sigma) as a chromogen and H_2_O_2 _(0.01%, v/v, Sigma).

The occupied area (μm^2^) of beta amyloid peptide immunoreactivity in the hippocampus cultures was calculated by means of a KS 400 image analyzer (Kontron, Zeiss, Germany) linked to a CCD 72 camera (Dage; MTI, Michigan City, Ind, USA) mounted on a Zeiss microscope (40× objective). Nine randomly chosen fields were considered for the quantification. The procedures have been described in detail elsewhere [18].

### Western blot analysis of protein aggregation

Cultured cells were homogenized in PBS, pH 7.4, containing 1% NP40, 0.5% sodium deoxycholate, 1%SDS, 1 mM EDTA, 1 mM EGTA and 1% protease inhibitor cocktail (Sigma). After centrifugation at 14000 rpm for 20 minutes, the resulting supernatant was fractionated by SDS-PAGE (10 μg of protein/lane) using a 12% tris-HCl gel at 100V for 1 h. Proteins were transferred to nitrocellulose membrane for 1 h at 100V.

Blots were incubated in blocking solution containing 5% milk/TBS-T during 1 h at room temperature followed by incubation with primary antibodies against alpha-synuclein (Abcam, 4D6, Ab1903) or hyperphosphorylated tau (Sigma, Ser 199/202, T6819) both were diluted 1/1000 in solution containing 3% milk/TBS-T, overnight at 4°C.

Horseradish peroxidase-conjugated secondary antibody incubations were performed at room temperature for 1 h with antibody anti-mouse 1/6000 (Amersham) or anti-rabbit 1/10000 (Amersham).

Development was done after 5-minute incubation with enhanced chemiluminescence reagent (Millipore) and exposure of membranes to ECL sensitive films (Hyperfilm ECL, Amersham Biosciences). After development, blots were incubated with anti-beta-actin antibody 1/1000 (Santa cruz, C4, sc-47778) during 1 h at room temperature, followed by horseradish peroxidase conjugated secondary antibody anti-mouse (Amersham) diluted 1/6000 for 1 hour also at room temperature and developed as previously described.

Density normalization was done by dividing the density of the bands relative to proteins of interest by beta-actin value. Films were quantified by optical densitometry using a system of image analysis (Imaging Research Inc., Canada, model M4/SK/ALU).

All the analyses were made using protein samples from control and treated cells that were fractionated in a same gel and transferred to a single membrane which was incubated with the antibody solution.

The presence of alpha-synuclein isoforms was confirmed in neonate and adult (6 months old) Wistar and Lewis rats. This was performed because the isoform of 14kDa did not appear in cultured cells from Lewis newborn rats. To this end, rats were euthanized and had their brains excised instantly to protein extraction of substantia nigra which was subjected to the same western blot method described previously for cell culture. In addition to the antibody from Abcam, an immunoglobulin against alfa-synuclein from Santa Cruz Biotechnology (1/500; cat. 7011R) was employed to confirm the pattern of isoforms present in neonates and adults. An assay of adsorption was performed by adding specific blocking peptide (1/100, Santa Cruz; cat. 7011P) to the solution containing the antibodies to confirm their specificity.

All the antibodies used in the present study were tested for specificity. In the case of immunocytochemistry it was done by incubating the sections with the secondary antibody only, and for western blot the control of specificity was tested in the presence of the blocking peptide. Furthermore, the antibodies are commercially available, their specificity are warranted by the manufacturer as well as they have been tested by other authors [19-21].

### Statistical analysis

Results were analyzed by unpaired Student's T test accessed through GraphPad Prism (GraphPad Software Inc., version 4.00, CA). A p-value ≤ 0.05 was considered to indicate statistically significant differences. Data are expressed as mean ± standard deviation (SD).

## Results

### Cell culture characterization

Quantification of cell cultures from the locus coeruleus and substantia nigra showed that 53% and 44%, respectively, of these cells expressed the enzyme tyrosine hydroxylase, essential for the synthesis of catecholamines (Table 1). Hippocampal cultures presented 53% of neurons labeled by MAP2 antibody (Table 1). This reveals that the cultures were suitable for studying substantia nigra, locus coeruleus and hippocampus cells.

**Table 1:** Cell culture characterization

	DAPI	TH or MAP2	% TH or MAP2 positive cells
**Locus coeruleus**	656	349	53.20
**Substantia nigra**	857	377	43.99
**Hippocampus**	1711	909	53.1

Quantification of the percentage of cells immunopositive to tyrosine hydroxylase (TH) or microtubule-associated protein 2 (MAP2, Hippocampus) in relation to the total number of cells, indicated by DAPI, in cells cultures of locus coeruleus, substantia nigra and hippocampus. Count was performed in 16 fields of each culture plate of a total of 4 plates per experiment.

### Effect of Rotenone on cell viability

**Figure 1:**
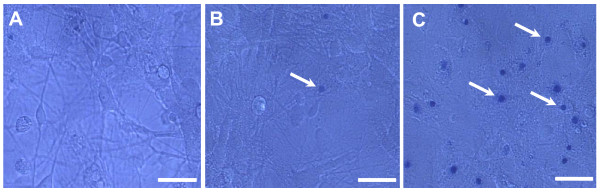
**Analysis of rotenone toxicity**. Photomicrographs illustrating the appearance of hippocampal cells treated with 0.5 nM (A), 1 nM (B) and 10 nM (C) of rotenone after addition of trypan blue in cell culture medium. Cells stained in blue (arrows) are under death process. Scale bar = 50 μm. Experiment was repeated three times. Cells from substantia nigra and locus coeruleus exhibited the same pattern of sensitivity.

Rotenone promoted cell death in a dose-dependent manner in cultures of hippocampus, substantia nigra and locus coeruleus. Rotenone at 0.5 nM seems not to be toxic to the hippocampal cells (Figure 1A). Incubation with 1 nM of rotenone during 48 h stained a few cells in blue (Figure 1B), however 10 nM of rotenone promoted a massive cell staining with trypan blue (Figure 1C). Cell cultures from substantia nigra and locus coeruleus exhibited the same pattern of sensitivity of hippocampal cells (data not shown).

### Presence of alpha-synuclein isoforms in newborn Lewis rats

**Figure 2:**
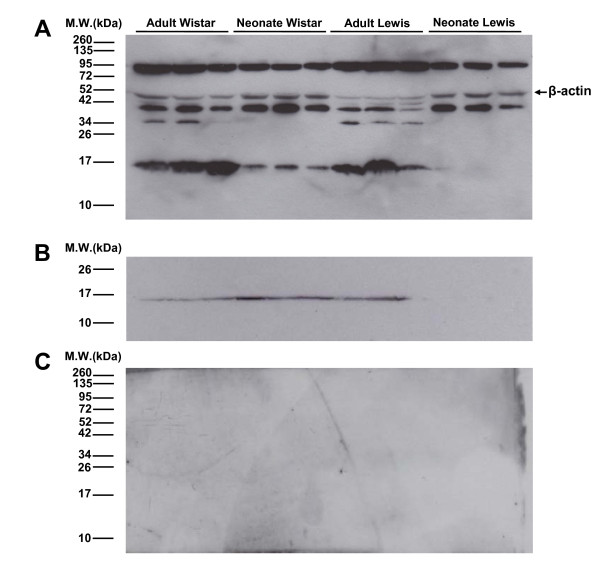
**Alpha-synuclein isoforms**. Western blot illustrating the isoforms of alpha-synuclein present in the substantia nigra of adult and newborn Wistar and Lewis rats using antibody from Abcam (A) and Santa Cruz (B), confirming the absence of the 14 kDa isoform of alpha-synuclein in neonate Lewis rats. The antibody from Abcam showed 3 isoforms of alpha-synuclein (90 kDa, 40 kDa and 14 kDa). Antibodies specificity was demonstrated by adding blocking peptide to the solution containing antibodies, which prevented the label of bands corresponding specifically to alpha-synuclein (C).

The presence of alpha-synuclein isoforms was analyzed in newborn and adult Wistar and Lewis rats because during pilot experiments the 14 kDa isoform never appeared in cultured cells from newborn Lewis rats. Figure 2A shows the pattern of isoforms labeling in newborn and adult rats from Wistar and Lewis strains using the antibody from Abcam. This confirms the absence of 14 kDa isoform in neonate Lewis rats and its presence in neonatal Wistar and adult rats of both strains. The isoforms of 40 kDa and 90 kDa are present in all ages and strains. Adult rats showed an additional band of 34 kDa (Figure 2A).

The same pattern of alpha-synuclein isoforms labeling was observed in neonate and adult rats when the antibody from santa cruz was employed (Figure 2B). The specificity of antibodies was demonstrated by the absence of labeling when incubating the antibodies with blocking peptide (Figure 2C).

### Alpha-synuclein protein aggregates

**Figure 3:**
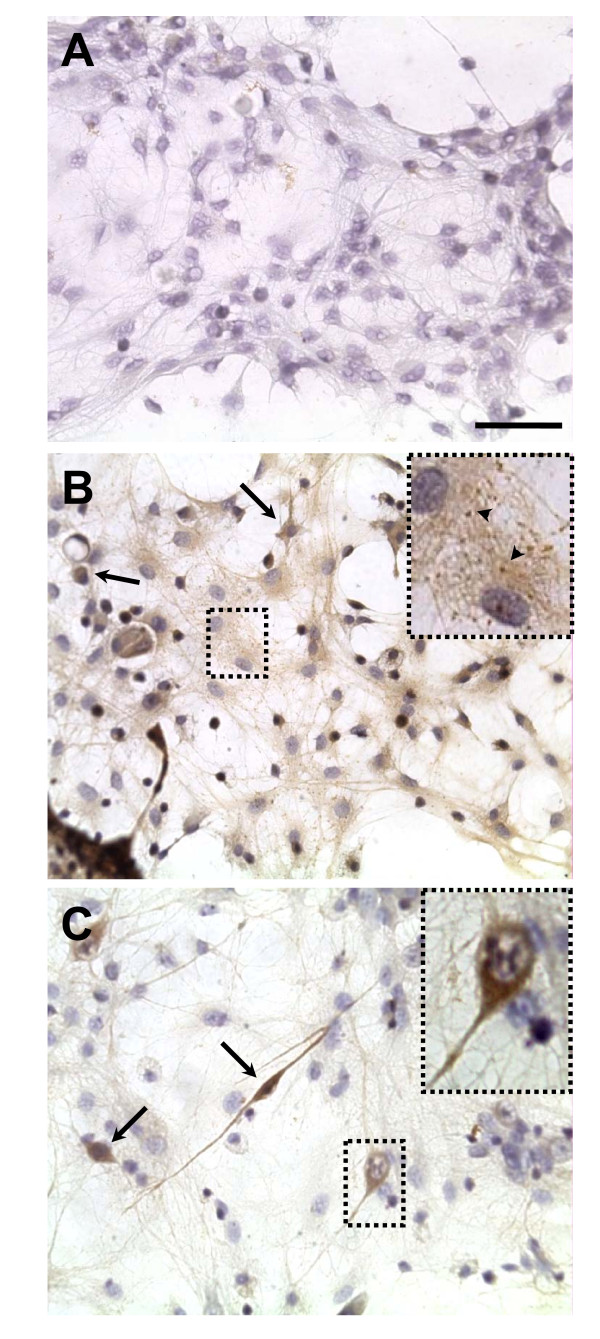
**Aggregation of alpha-synuclein after rotenone exposure**. Photomicrographs illustrating the immunoreactivity of alpha-synuclein in cultured cells of the locus coeruleus exposed to DMSO (A) or 0.5 nM rotenone (B) and hippocampal cells exposed to 1 nM of rotenone (C) for 48 hours. Arrows indicate cells with dense cytoplasm aggregates of alpha-synuclein. Arrowheads indicate sites of alpha-synuclein aggregation (detail in B). Cells were counterstained with hematoxylin (purple) after immunoreaction. Scale bar = 50 μm.

Immunocytochemical staining using anti-alpha-synuclein antibody revealed that rotenone at 0.5 nM or 1 nM promoted aggregation of alpha-synuclein in cell bodies and extensions of the locus coeruleus and hippocampus as compared to DMSO-exposed cells (Figure 3). Besides the protein aggregation, exposure of hippocampal cells to 1 nM of rotenone promoted apparent retraction of neuronal processes as well as reduced the total number of cells.

**Figure 4:**
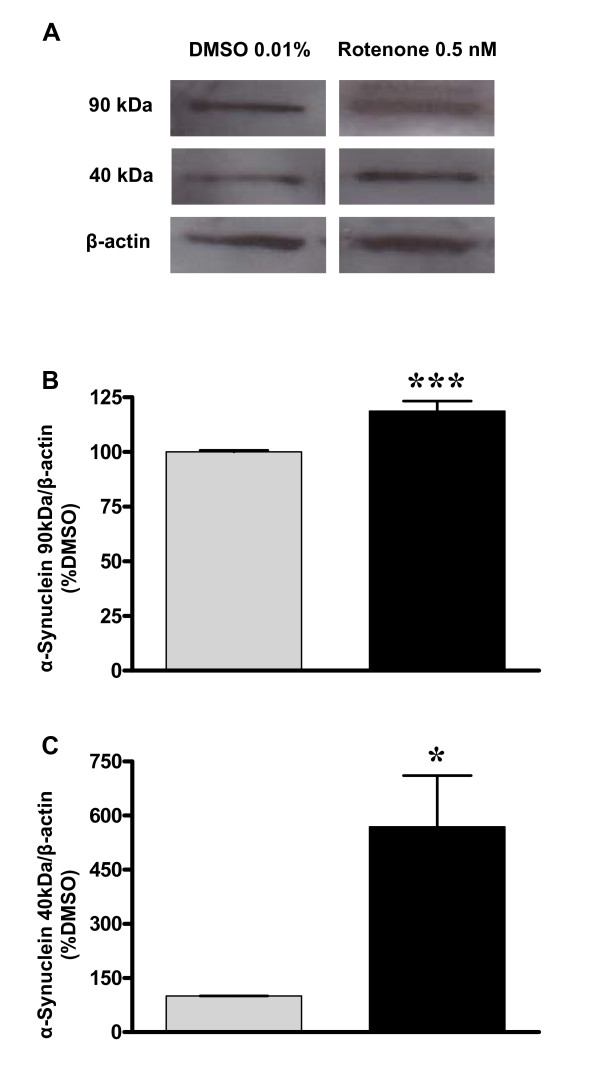
**Quantification of alpha-synuclein aggregation after rotenone exposure**. Expression of alpha-synuclein isoforms of 90 kDa and 40 kDa in cell cultures of substantia nigra following incubation with DMSO or 0.5 nM of rotenone during 48 h. Normalization was performed by beta-actin (43 kDa) signal. Data are shown as percent of control (DMSO) ± S.D. * p < 0.05 as compared to control (DMSO), according to Student's t-test. The experiment was repeated twice, which were run in triplicates.

Western blot experiments were conducted to confirm and quantify alpha-synuclein aggregation in substantia nigra cells. In that assay there were two specific bands corresponding to alpha-synuclein one of 90 kDa and another of 40 kDa, which were up-regulated by 25% and 400%, respectively, following 0.5 nM of rotenone as compared to DMSO-exposed cells (Figure 4).

### Hyperphosphorylation of tau

**Figure 5:**
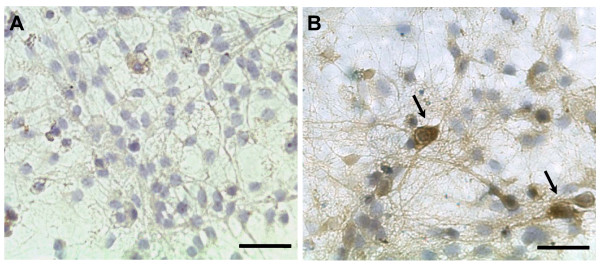
**Increased hyperphosphorylation of tau after rotenone exposure**. Photomicrographs illustrating the immunoreactivity of hyperphosphorylated tau in cultured cells of hipoccampus exposed to DMSO (A) or 0.5 nM of rotenone (B) for 48 hours. Arrows indicate sites of hyperphosphorylated tau, possibly aggregated, distributed throughout cytoplasm and neurites. Scale bar = 50 μm.

**Figure 6:**
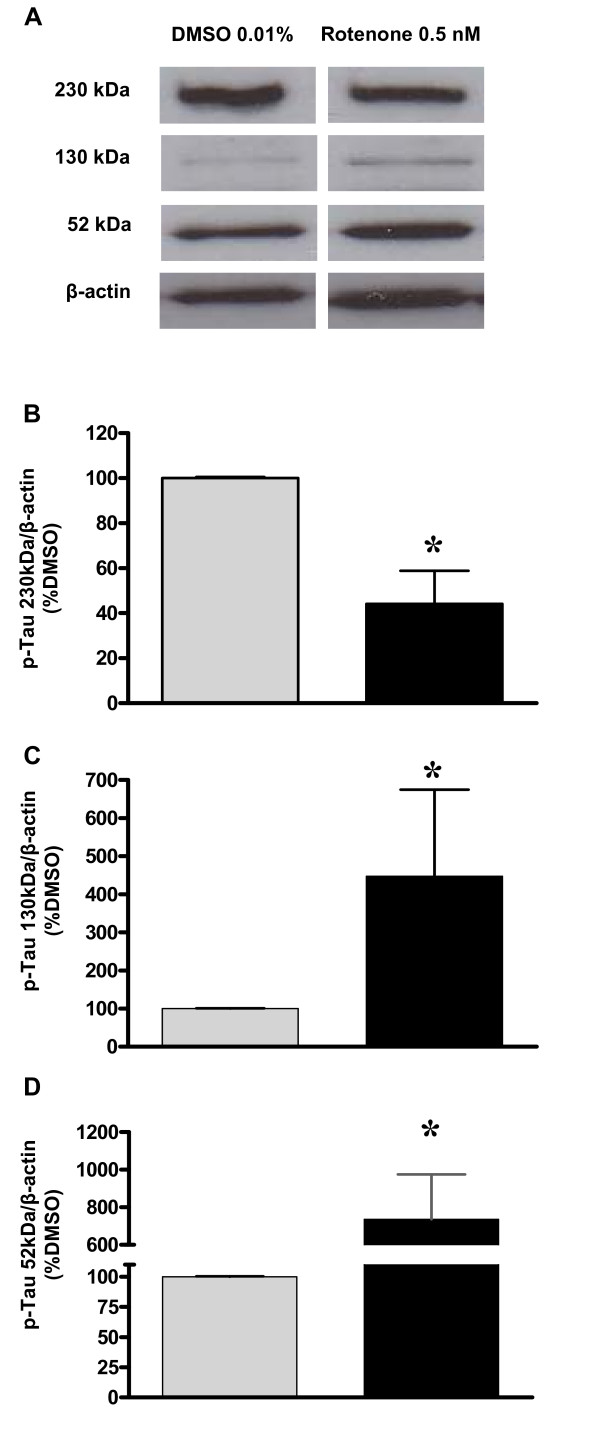
**Quantification of hyperphosphorylated tau after rotenone exposure**. Illustrative images of the pattern of bands corresponding to hyperphosphorylated tau in hippocampal cells exposed to 0.5 nM of rotenone or DMSO for 48 hours (A). The 230 kDa bands are considered the insoluble fraction of hyperphosphorylated tau, which seems to be reduced after rotenone (B), the 130 kDa bands are considered as oligomers of hyperphosphorylated tau (C) and 52-kDa bands as monomers of hyperphosphorylated tau (D), which are increased after prolonged exposure to low concentration of rotenone. Normalization was performed by beta-actin (43 kDa) signal. Values are shown as percentage of control (DMSO) ± S.D. * p < 0.05 as compared to control (DMSO), according to Student's t-test. The experiment was repeated twice, which were run in triplicates.

Cells from the hippocampus were stained with anti-hyperphosphorylated tau antibody demonstrated that 0.5 nM of rotenone also promotes increase in hyperphosphorylation of tau protein in cell body and extensions (Figure 5). Densitometric analysis of the immunoblots corroborate with immunocytochemical experiments showing that isoforms of hiperphosphorylated tau of 52 and 130 kDa increase their expression demonstrating possibly the hyperphosphorylated tau in hippocampal cells culture after exposure to 0.5 nM of rotenone for 48 hours (Figure 6). The 230 kDa isoform showed an unexpected significant decrease in expression, which may reveal that, at this level of hyperphosphorylation, tau is not aggregated yet.

### Identification of beta-amyloid protein aggregates

**Figure 7:**
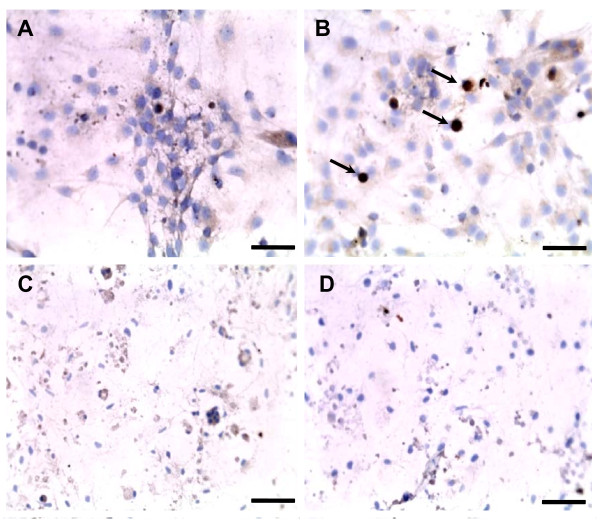
**Aggregation of beta-amyloid peptide after rotenone exposure**. Photomicrographs of cultured hippocampal cells exposed to DMSO (A) or rotenone at concentrations of 1 nM (B), 10 nM (C) and 25 nM (D) for 48 hours, illustrating the profile of beta-amyloid immunoreactivity. Arrows indicate the aggregates (B). Rotenone at high concentrations promoted intense cell death demonstrated by the fragmented and retracted nuclei (C and D), there is no evidence of beta-amiloid aggregation. Nuclei were stained with hematoxylin. Scale bar = 50 μm.

The analysis of beta-amyloid aggregation was done by immunocytochemistry which demonstrates that the exposure to 1 nM of rotenone was effective in developing protein aggregates containing that peptide as compared to cells exposed to DMSO (Figures 7A and 7B). Higher concentrations of rotenone (10 and 25 nM) promoted nuclear retraction and fragmentation, as well as decrease in cell extensions (Figures 7C and 7D) demonstrating that high concentrations of the pesticide probably leads cells to death before the aggregation of beta-amyloid.

**Figure 8:**
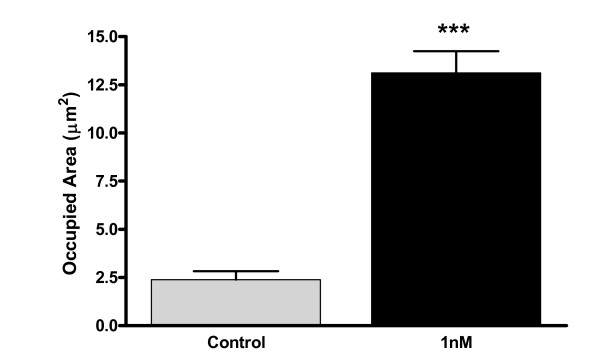
**Quantification of beta-amyloid aggregation after rotenone exposure**. Area occupied by beta-amyloid immunopositive aggregates in hippocampal cells exposed to 1 nM of rotenone during 48 h. Values are shown as mean±SD ***p < 0.0001 as compared to control cells (culture medium) from the same strain according to Student's t-test. The experiment was repeated twice.

Quantification of area occupied by beta-amyloid imunopositive aggregates showed a significant increase after exposure to 0.5 nM of rotenone for 48 hours (13.12 ± 1.12) as compared to the control (2.39 ± 0.43) (Figure 8).

## Discussion

To our knowledge this is the first report of *in vitro *protein aggregation promoted by the inhibition of mitochondrial respiratory chain complex I, these findings are of special interest for studying proteins involved in neurodegeneration such as alpha-synuclein, hyperphosphorylated tau and beta-amyloid.

High concentrations of rotenone (over 1 nM) lead to cell death prior to protein aggregation as demonstrated by trypan blue stain and amyloid beta immunocytochemistry independently of the region studied. Rotenone is an inhibitory drug of the mitochondrial respiratory chain complex I. However, low concentration of this compound (0.5 and 1 nM) is able to promote constitutive protein aggregation.

Many studies have employed *in vivo *and *in vitro *models to evaluate the neurodegeneration process. These models of neurodegeneration may involve the use of drugs such as 6-hydroxydopamine (6-OHDA), 1-methyl-4-phenyl-1,2,3,6-tetrahydropyridine (MPTP) and rotenone [22], or overexpression of mutant genes in rats, mice and cell lineages [23-26]. Although these models are relevant to understand some aspects involved neurodegeneration, there is still lack of information about the events that cause constitutive protein aggregation and its role during the neurodegenerative processes. Recently De-Paula and colleagues [27] described that the inhibition of phospholipase A2 led to hyperphosphorylation of tau in primary cell culture, but the aggregation of other proteins such as alpha-synuclein and beta amyloid was not analyzed.

The use of primary cell culture, including neurons and glia, approximates the *in vitro *model and the physiological situation encountered in the brain. Recent evidences showed that dopaminergic death may be preceded by glial disfunction [28,29], as well as the interaction between neurons and astrocytes is of relevance to the course of neurodegeneration [30]. In view of this, we believe that the model using neurons and glial cells are more related to the real neuropathology than studying purified cell cultures.

The present study demonstrate that there is protein aggregation in condition of mitochondrial stress and proteasome inhibition which is a common feature encountered during aging [31]. Thus, our model has some advantages over the genetic and transfection models since the aggregation is mainly sporadic and formed by constitutive proteins, similar to what occurs during ageing and neurodegeneration.

To validate our study we performed an assay to characterize the presence of alpha-synuclein in neonatal and adult rats. In this study we demonstrated that the isoform of 14 kDa of alpha-synuclein, which is the most studied isoform of this protein, is not present in neonatal Lewis rats but is expressed in the adult age. Since cultures were made using newborn Lewis rats there was not label of the 14 kDa isoform alpha-synuclein in aggregation studies. The use of Lewis rats in our experiments was important because of their exclusive susceptibility to form protein aggregates after treatment with rotenone in *in vivo *experiments [32,33].

Rotenone was able to induce alpha-synuclein aggregation, which is of interest for studying neurodegeneration associated to protein aggregation. Not only the aggregation itself is related to neurodegeneration, the imbalance of neurotransmission may trigger neurodegenerative mechanisms, evidences indicate that alpha-synuclein is encountered in association with the presynaptic vesicle pool [34] and the aberrant association between alpha-synuclein and rab proteins [35] may be of relevance in the study of cellular stress that leads to neurodegenarative disorders.

Hyperphosphorylation of tau protein is a condition that disrupts the intracellular trafficking impairing neurotransmission in association with formation of intracellular inclusion of paired helical filaments. In the present study we demonstrated that rotenone may be involved in hyperphosphorylation of tau protein as demonstrated by the increase in optical density of 52 kDa subunit. The fraction of 130 kDa is increased in rotenone-treated cells demonstrating a possible mechanism of aggregation that occurs in low concentrations of rotenone. The 130 kDa band may correspond to small aggregates (dimerization/trimerization) of hyperphosphorylated tau, as previously demonstrated [36,37].The presence of tau hyperphosphorylated in control groups is apparently normal in neonatal rats and has been described by Goedert and colleagues [38].

Beta-amyloid plaques were found in cells from hippocampus after incubation during 48 h with rotenone. High concentrations of the drug is toxic leading to cell death before protein aggregation, which is devoid to the high toxicity. Furthermore, endogenous increase of amyloid beta peptide production preceeds hyperphosphorylation of tau [39], which may be of interest to evaluate the mechanism of protein aggregation.

Besides the well known effects of rotenone in inhibiting mitochondrial complex I, the pesticide is also able to impair protein degradation by interfering with the ubiquitin-proteasome-system [40], in view of this the aggregation seen in the present study may be also in response to the deficit of the cellular systems of protein degradation in addition to the increase in oxidative stress. Actually Branco and co-workers demonstrated the cross-talk between mitochondria and proteasome during the pathogenesis of Parkinson's disease [41]. The effect of rotenone over the impairment of the proteasome is being conducted as a parallel study in our laboratory.

## Conclusions

In conclusion this study demonstrated a new method to study constitutive protein aggregation *in vitro *by the exposure of neonatal cultured cells of Lewis rats to low concentrations of rotenone, which may be of relevance to understand the mechanisms that lead to idiopathic neurodegeneration.

## Competing interests

The authors declare that they have no competing interests.

## Authors' contributions

RSC carried out cell culture characterization, cell death assay, immunocytochemistry of alpha-synuclein and western blot experiments. TQM performed beta-amyloid immunocytochemistry in cultured cells made by herself. SAM carried out cell cultures and tau immunocytochemistry. MFRF conceived of the study, designed and coordinated the experiments. All authors analyzed the results, participated of manuscript draft, read and approved the final manuscript.

## References

[B1] Ross CA Poirier MA Opinion: What is the role of protein aggregation in neurodegeneration? Nature reviews 2005 6 11 891 898 10.1038/nrm1742 16167052

[B2] Dayan AD Quantitative histological studies on the aged human brain. I. Senile plaques and neurofibrillary tangles in "normal" patients Acta neuropathologica 1970 16 2 85 94 10.1007/BF00687663 4919692

[B3] Jager W Bethlem J The distribution of Lewy bodies in the central and autonomic nervous systems in idiopathic paralysis agitans J Neurol Neurosurg Psychiatry 1960 23 283 290 10.1136/jnnp.23.4.283 13711997 PMC497426

[B4] Ohama E Ikuta F Parkinson's disease: distribution of Lewy bodies and monoamine neuron system Acta neuropathologica 1976 34 4 311 319 10.1007/BF00696560 179263

[B5] Oyanagi K Wakabayashi K Ohama E Takeda S Horikawa Y Morita T Ikuta F Lewy bodies in the lower sacral parasympathetic neurons of a patient with Parkinson's disease Acta neuropathologica 1990 80 5 558 559 10.1007/BF00294619 2251914

[B6] Kakita A Takahashi H Homma Y Ikuta F Lewy bodies in the cerebellar dentate nucleus of a patient with Parkinson's disease Pathology international 1994 44 12 878 880 10.1111/j.1440-1827.1994.tb01688.x 7866573

[B7] Wakabayashi K Takahashi H Neuropathology of autonomic nervous system in Parkinson's disease Eur Neurol 1997 38 2 7 10.1159/000113469 9387796

[B8] Takahashi RH Nam EE Edgar M Gouras GK Alzheimer beta-amyloid peptides: normal and abnormal localization Histology and histopathology 2002 17 1 239 246 11813874 10.14670/HH-17.239

[B9] Bourgeat P Chetelat G Villemagne VL Fripp J Raniga P Pike K Acosta O Szoeke C Ourselin S Ames D Beta-amyloid burden in the temporal neocortex is related to hippocampal atrophy in elderly subjects without dementia Neurology 2010 74 2 121 127 10.1212/WNL.0b013e3181c918b5 20065247

[B10] Jellinger KA Recent advances in our understanding of neurodegeneration J Neural Transm 2009 116 9 1111 1162 10.1007/s00702-009-0240-y 19707851

[B11] Sherer TB Kim JH Betarbet R Greenamyre JT Subcutaneous rotenone exposure causes highly selective dopaminergic degeneration and alpha-synuclein aggregation Experimental neurology 2003 179 1 9 16 10.1006/exnr.2002.8072 12504863

[B12] Dukes AA Korwek KM Hastings TG The effect of endogenous dopamine in rotenone-induced toxicity in PC12 cells Antioxidants & redox signaling 2005 7 5-6 630 638 10.1089/ars.2005.7.630 15890007

[B13] Sherer TB Betarbet R Kim JH Greenamyre JT Selective microglial activation in the rat rotenone model of Parkinson's disease Neuroscience letters 2003 341 2 87 90 10.1016/S0304-3940(03)00172-1 12686372

[B14] Chou AP Li S Fitzmaurice AG Bronstein JM Mechanisms of rotenone-induced proteasome inhibition Neurotoxicology 2010 31 4 367 372 10.1016/j.neuro.2010.04.006 20417232 PMC2885979

[B15] Huang J Hao L Xiong N Cao X Liang Z Sun S Wang T Involvement of glyceraldehyde-3-phosphate dehydrogenase in rotenone-induced cell apoptosis: relevance to protein misfolding and aggregation Brain research 2009 1279 1 8 10.1016/j.brainres.2009.05.011 19445904

[B16] Silva JM Wong A Carelli V Cortopassi GA Inhibition of mitochondrial function induces an integrated stress response in oligodendroglia Neurobiology of disease 2009 34 2 357 365 10.1016/j.nbd.2009.02.005 19233273

[B17] Kivell BM McDonald FJ Miller JH Method for serum-free culture of late fetal and early postnatal rat brainstem neurons Brain research 2001 6 3 91 99 11223407 10.1016/s1385-299x(00)00037-4

[B18] Zoli M Zini I Agnati L Guidolin D Ferraguti F Fuxe K Aspects of neuronal plasticity in the central nervous system. I. Computer-assisted image analysis methods Neurochem Int 1990 16 383 418 10.1016/0197-0186(90)90002-B 20504581

[B19] Carrettiero DC Hernandez I Neveu P Papagiannakopoulos T Kosik KS The cochaperone BAG2 sweeps paired helical filament- insoluble tau from the microtubule J Neurosci 2009 29 7 2151 2161 10.1523/JNEUROSCI.4660-08.2009 19228967 PMC2768429

[B20] Marksteiner J Humpel C Beta-amyloid expression, release and extracellular deposition in aged rat brain slices Molecular psychiatry 2008 13 10 939 952 10.1038/sj.mp.4002072 17712316

[B21] Vogiatzi T Xilouri M Vekrellis K Stefanis L Wild type alpha-synuclein is degraded by chaperone-mediated autophagy and macroautophagy in neuronal cells The Journal of biological chemistry 2008 283 35 23542 23556 10.1074/jbc.M801992200 18566453 PMC2527094

[B22] Schmidt WJ Alam M Controversies on new animal models of Parkinson's disease pro and con: the rotenone model of Parkinson's disease (PD) Journal of neural transmission 2006 70 273 276 17017541

[B23] Gotz J Streffer JR David D Schild A Hoerndli F Pennanen L Kurosinski P Chen F Transgenic animal models of Alzheimer's disease and related disorders: histopathology, behavior and therapy Molecular psychiatry 2004 9 7 664 683 15052274 10.1038/sj.mp.4001508

[B24] Hattori N Sato S Animal models of Parkinson's disease: similarities and differences between the disease and models Neuropathology 2007 27 5 479 483 10.1111/j.1440-1789.2007.00842.x 18018484

[B25] Lee HJ Shin SY Choi C Lee YH Lee SJ Formation and removal of alpha-synuclein aggregates in cells exposed to mitochondrial inhibitors The Journal of biological chemistry 2002 277 7 5411 5417 10.1074/jbc.M105326200 11724769

[B26] Schule B Pera RA Langston JW Can cellular models revolutionize drug discovery in Parkinson's disease? Biochim Biophys Acta 2009 1792 11 1043 1051 19733239 10.1016/j.bbadis.2009.08.014

[B27] De-Paula VJ Schaeffer EL Talib LL Gattaz WF Forlenza OV Inhibition of phospholipase A2 increases tau phosphorylation at Ser214 in embryonic rat hippocampal neurons Prostaglandins, leukotrienes, and essential fatty acids 2010 82 1 57 60 10.1016/j.plefa.2009.07.006 19726172

[B28] Hirsch EC Hunot S Neuroinflammation in Parkinson's disease: a target for neuroprotection? Lancet neurology 2009 8 4 382 397 10.1016/S1474-4422(09)70062-6 19296921

[B29] Tansey MG Goldberg MS Neuroinflammation in Parkinson's disease: its role in neuronal death and implications for therapeutic intervention Neurobiology of disease 2010 37 3 510 518 10.1016/j.nbd.2009.11.004 19913097 PMC2823829

[B30] Mullett SJ Hinkle DA DJ-1 knock-down in astrocytes impairs astrocyte-mediated neuroprotection against rotenone Neurobiology of disease 2009 33 1 28 36 10.1016/j.nbd.2008.09.013 18930142 PMC2638760

[B31] Boveris A Navarro A Brain mitochondrial dysfunction in aging IUBMB Life 2008 60 5 308 314 10.1002/iub.46 18421773

[B32] Testa CM Sherer TB Greenamyre JT Rotenone induces oxidative stress and dopaminergic neuron damage in organotypic substantia nigra cultures Molecular Brain Research 2005 134 1 109 118 10.1016/j.molbrainres.2004.11.007 15790535

[B33] Sherer TB Betarbet R Testa CM Seo BB Richardson JR Kim JH Miller GW Yagi T Matsuno-Yagi A Greenamyre JT Mechanism of toxicity in rotenone models of Parkinson's disease J Neurosci 2003 23 34 10756 10764 14645467 10.1523/JNEUROSCI.23-34-10756.2003 PMC6740985

[B34] Murphy DD Rueter SM Trojanowski JQ Lee VM Synucleins are developmentally expressed, and alpha-synuclein regulates the size of the presynaptic vesicular pool in primary hippocampal neurons J Neurosci 2000 20 9 3214 3220 10777786 10.1523/JNEUROSCI.20-09-03214.2000 PMC6773130

[B35] Dalfo E Barrachina M Rosa JL Ambrosio S Ferrer I Abnormal alpha-synuclein interactions with rab3a and rabphilin in diffuse Lewy body disease Neurobiology of disease 2004 16 1 92 97 10.1016/j.nbd.2004.01.001 15207266

[B36] Blard O Frebourg T Campion D Lecourtois M Inhibition of proteasome and Shaggy/Glycogen synthase kinase-3beta kinase prevents clearance of phosphorylated tau in Drosophila Journal of neuroscience research 2006 84 5 1107 1115 10.1002/jnr.21006 16878320

[B37] Shelton SB Johnson GV Tau and HMW tau phosphorylation and compartmentalization in apoptotic neuronal PC12 cells Journal of neuroscience research 2001 66 2 203 213 10.1002/jnr.1212 11592115

[B38] Goedert M Jakes R Crowther RA Six J Lubke U Vandermeeren M Cras P Trojanowski JQ Lee VM The abnormal phosphorylation of tau protein at Ser-202 in Alzheimer disease recapitulates phosphorylation during development Proceedings of the National Academy of Sciences of the United States of America 1993 90 11 5066 5070 10.1073/pnas.90.11.5066 8506352 PMC46655

[B39] Amadoro G Corsetti V Ciotti MT Florenzano F Capsoni S Amato G Calissano P Endogenous Abeta causes cell death via early tau hyperphosphorylation Neurobiol Aging 2009 in press 19628305 10.1016/j.neurobiolaging.2009.06.005

[B40] Wang XF Li S Chou AP Bronstein JM Inhibitory effects of pesticides on proteasome activity: implication in Parkinson's disease Neurobiology of disease 2006 23 1 198 205 10.1016/j.nbd.2006.02.012 16626962

[B41] Branco DM Arduino DM Esteves AR Silva DF Cardoso SM Oliveira CR Cross-talk between mitochondria and proteasome in Parkinson's disease pathogenesis Frontiers in aging neuroscience 2010 2 17 20577640 10.3389/fnagi.2010.00017 PMC2890153

